# 

**Published:** 1887-11-05

**Authors:** 


### NOTICE TO ADVERTISERS.

THE scale of charges for small prepaid Advertisements—Situations Wanted, Situations Vacant, &c., &c., is as follows:—

| | s. | d. |
|---|---|---|
| One insertion of three lines | 1 | 0 |
| Three " " " | 2 | 6 |
| Per line afterwards | 0 | 6 |

*A line averages ten words.*

All copy for Advertisements must be sent to A. P. WATT, 2, PATERNOSTER SQUARE, E.C., by first post on the Tuesday *preceding* publication.

### NOTICE TO CORRESPONDENTS.

All correspondence must be written on one side of the paper only. We cannot undertake to return MSS. not used.

**Communications,** whether intended for insertion or for private information, must be authenticated by the names and addresses of the writers, not necessarily for publication.

**Local papers** containing reports or news paragraphs **should be marked.**

It is especially requested that early intelligence of **local events of interest,** or which it may be thought desirable to bring under notice, may be sent direct to the Editor.

Communications respecting editorial matters should be addressed to the Editor, Norfolk House, Norfolk Street, Strand, London, W.C.

### THE STUDENTS' COLUMN.

### ROYAL COLLEGE OF SURGEONS OF ENGLAND.

### PRIMARY EXAMINATION, October, 1887.

PASS LIST.

*Arkle, George Manning, Liverpool; †Bashall, Charles Edward, St. Thomas's; Bayley, Joseph Herbert, Edinburgh; *Bickerstaff, George Roger, St. Mary's; Booth, Richard Dalby, Edinburgh; Campion, George Alexander, St. Thomas's; †Daly, James Robert, Manchester; Dowding, William Frederick Charles, St. George's; †Dudgeon, Robert Henry Beauchamp, Liverpool; Eccles, Robert Burton, St. Thomas's; Findlay, Harry, Glasgow; *Gray, Thomas, Edinburgh; †Griffith, John Samuel, Bristol; Gyton, Walter George, Manchester; Hawes, Godfry Charles Browne, St. George's; †Hopps, Thomas, Manchester; †Kelly, Charles Ernest Mackenzie, Manchester; Lupton, James, Manchester; McQueen, Chas. Alexander Simpson, Pennsylvania; Nolan, Joseph Adam, Edinburgh; Rahn, Ernest William, Edinburgh; †Ram, Bhagat, Lahore; †Rich, Mark Ringwood, London; †Ryall, Edward Canny, Dublin; Skipton, Stacy Charles, St. Thomas's; Smith, Colvin Burslem, Newcastle; †Springett, Edmund, St. Thomas's; Standring, Charles Turner, King's College; Thistle, William George, Guy's; Tyson, Edward, St. Bartholomew's; Walker, Harold Albert, Manchester; *Welch, Walter Barrows, St. Bartholomew's; Williams, Frederick Edgar, Guy's; †Winnett, Frederick, Toronto; Woodcock, Arthur Ernest, Leeds; †Yeoman, George, Cambridge.

* Passed in Anatomy only. † Passed in Physiology only.

### ROYAL COLLEGE OF PHYSICIANS OF LONDON.

### PRIMARY EXAMINATION, October, 1887.

PASS LIST.—CHEMISTRY.

Bates, James Curling, Guy's; Best, William Harris, London; Bishop, Reginald Wm. Snowden, Leeds; Booth, Oscar Milner, Leeds; Buck, Walter, Manchester; Burland, Herbert, Manchester; Burrows, Charles William Grimes, Charing Cross; Burrows, Walter Horncastle, Charing Cross; Cotton, William Mitchell, University College; Cox, Arthur Brooks, Middlesex; Craig, William Wallace, University College; Crickett, Henry Halkett, St. George's; Dawber, John Henry, Middlesex; Evans, Thomas Henry Fencott, St. Bartholomew's; Eyton-Jones, John Arthur, Liverpool; Foster, Franz Forester Foster, Guy's; Greenwood, George Spencer, Leeds; Grosvenor, Alfred Augustus, Guy's; Handcock, George, Leeds; Hebblethwaite, Rhodes, Leeds; Kent, Robert Thomas, Edinburgh; Lambert, Edwin, Leeds; Lander, Francis Joseph, London; McCrea, Benjamin Henry Edward, London; Maberley, John, Middlesex; Metcalfe, George Edward Gibson, St. Bartholomew's; Murray-Aynsley, John Henry, St. George's; Pritchett, Sidney Isaac, Birmingham; Seymour, George, Middlesex; Smith, Henry Archbold, Leeds; Steele, Ernest Arthur, Liverpool; Stephenson, Owen Taunton, Liverpool; Stevens, Charles Henry, University College; Stevens, William Edward, Bristol; Walker, Harold Albert, Manchester; Watkins, William James, Bristol; Williams, Ernest Graham Hamilton, Charing Cross; Williams, Richard Henry, University College; Woollett, Sidney Winslow, King's College.

PASS LIST.—MATERIA MEDICA AND PHARMACY.

Alexander, Charles Robert Barry, St. Bartholomew's; Ash, James, Charing Cross; Best, William Harris, London; Bishop, Reginald William Snowden, Leeds; Broadhurst, William James, University College; Burland, Herbert, Manchester; Burrows, Charles William Grimes, Charing Cross; Burrows, Walter Horncastle, Charing Cross; Bynoe, Charles Augustus, University College; Callender, Eustace Maude, St. Mary's; Caudwell, Francis Bernard Henry, Charing Cross; Clayton, Rupert Wilberforce, St. Mary's; Collins, Frank, University College; Cox, Arthur Brooks, Middlesex; Crickett, Henry Halkett, St. George's; Cullen, Ebenezer, Guy's; Evans, Thomas Henry Fencott, St. Bartholomew's; Eyton-Jones, John Arthur, Liverpool; Finnie, John Ellison, Liverpool; Foster, Franz Forester Foster, Guy's; Gott, Henry, Leeds; Handcock, George, Leeds; Harper, Charles Randle, Middlesex; Howell, Arthur Henry, London; Johnson, Benjamin Pitt, Liverpool; Kent, Robert Thomas, Edinburgh; Leakey, Charles Montague, London; McCrea, Benjamin Henry Edward, London; Mansbridge, Josiah, Charing Cross; Metcalfe, George Edward Gibson, St. Bartholomew's; Miers, Arthur, Leeds; Morgan, Morgan John, Guy's; Morris, Arthur Levi, Guy's; Murray-Aynsley, John Henry, St. George's; Paget, Peter, Guy's; Pearson, James, University College; Pettitt, William Brackenbridge, Leeds; Pritchett, Sidney Isaac, Birmingham; Rouw, Robert Wynne, Guy's; Row, Linfield Elfe, London; Rubel, John Leopold, King's College; Seymour, George, Middlesex; Smith, Stephen Francis, London; Steele, Ernest Arthur, Liverpool; Stevens, William Edward, Bristol; Turnor, Philip Watson, St. George's; Valentine, Thos. Harcourt Ambrose, St. Bartholomew's; Verano, Louis Lawrence, St. Bartholomew's; Vicars, Frederick George, Guy's; Walker, Harold Albert, Manchester; Watkins, William James, Bristol; Williams, John Cobden, Liverpool; Williams, Richard Henry, University College; Woollett, Sidney Winslow, King's College.

### ROYAL COLLEGE OF PHYSICIANS OF LONDON AND THE ROYAL COLLEGE OF SURGEONS OF ENGLAND.

### FIRST EXAMINATION.

PART I.—CHEMISTRY, INCLUDING CHEMICAL PHYSICS.

TUESDAY, OCTOBER 18, 1887.

The following were the QUESTIONS given, not more than Four to be answered:—

SECTION I.—1. Define what is meant by Specific Gravity. How would you determine the specific gravity of a solid heavier than water, of one lighter than water, and of a liquid? 2. An electric current is passed through a solution of Copper Sulphate, describe what happens. Calculate the amount of Zinc Sulphate found in the battery when 1 grm. of copper has been deposited (Cu=63, Zn=65).

SECTION II.—3. Describe what is meant by the Allotropic Forms of an Element? How are the allotropic forms of Oxygen, Carbon, and Sulphur prepared? 4. One of the principal sources of Magnesium Salts is the Sulphate; how can you obtain from this salt the Carbonate, Oxide, and Metallic Magnesium? 5. How can the Permanganate of Potash be prepared from the Black Oxide of Manganese? Give its composition and properties.

SECTION III.—6. How is Cane-sugar prepared from the Sugar-cane? Give its composition and properties. Describe how Ethylic Alcohol can be obtained from it. 7. What is the usual process of manufacture of Oxalic Acid? State the composition and pr perties of the Acid. What changes occur when it is (*a*) heated on Platinum Foil, (*b*) heated with strong Sulphuric Acid, and (*c*) mixed with a solution of Caustic Soda? 8. How is Iodoform prepared? Give its composition, and describe its properties. How would you prove that Iodine is one of its constituents?

The following are the names of those who passed: Addison, Christopher, St. Bartholomew's; Alderson, Frederick Herbert, Middlesex; Allen, Andrew, Charing Cross; Allott, John Ernest Cecil, Sheffield; Baker, Nugent Mander, St. Bartholomew's; Barr, Valentine Herbert, Guy's; Bartlett, Frank Whinfield, University College; Bastard, Edward Roger, St. Bartholomew's; Beetham, Herbert Arthur, Leeds; Bicket, John Hugh, London; Burton, Thomas, Private; Cardinall, Charles Daking, St. Bartholomew's; Collings, Edward Beresford, Leeds; Cooper, Arthur Tanner, Private; Cornish, Edward, Guy's; Cross, Henry, Sheffield; Dale, Cuthbert Bracy, St. Bartholomew's; Davies, David Livingstone, Manchester; Deas, Frank, University College; De Castro, George, St. George's; De Renzi, Henry Carter Castriot, Westminster; Edwards, Philip Hugh, Birmingham; Everett, Ernest William, St. Bartholomew's; Exley, William Walker, Leeds; Fearnley, Jonathan, Leeds; Fincham, Ernest Charles, St. Bartholomew's; Forman, George Herbert, St. Bartholomew's; Foulkes, Thomas Howard, St. Bartholomew's; Francis, Harvey, St. Mary's; Francis, Louis Arthur, St. Mary's; Freese, John Henry, London; Goldney, Thomas William, Charing Cross; Greaves, Thomas Neville, King's College; Green, Arthur Stanley, St. Thomas's; Green, Percy Andrew, London; Hadaway, James Herbert, Middlesex; Haines, Arthur, St. Thomas's; Hall, William, Manchester; Handcock, William, Leeds; Headridge, David, Manchester; Herbert, Alfred William Corbyn, St. Mary's; Higgs, Ernest William Mules, Charing Cross; Hinde, William Cathcart, Middlesex; Hinks, Alfred Grosvenor, St. Mary's; Holt, Robert, Manchester; Howle, Walter Cresswell, Birmingham; Humphrey, Arthur Dumville, St. Bartholomew's; Ingram, Reginald Edward Tierney, Guy's; Jones, George Reginald, Liverpool; Knight, Reginald Brodnax, King's College; Knowles, Ralph, Manchester; Leonard, Robert Cecil, Bristol; Lilley, George William, Leeds; Lister, Septimus Rayner, St. Bartholomew's; Mackeson, Guy, Guy's; Madge, Edward Douglas, Middlesex; Maggs, Oliver Henry Arthur, Charing Cross; Manlove, James Ernest, St. Bartholomew's; Martin, Charles Rudinge, St. George's; Maw, Henry Solomon, St. Bartholomew's; Moore, Reginald Devereux, St. Mary's; Nunes, Herbert Fitz-Stephen, St. Mary's; O'Brien, Murrough Charles, St. Mary's; Oglesby, Frederick Loveday, Leeds; Orr, Walter Hood, St. Bartholomew's; Perkins, Henry Campbell, King's College; Pierce, William Arthur, Liverpool; Pollitt, William Edgar, Leeds; Powys, Henry Lionel, London; Prance, George Frederick Charles, St. Thomas's; Preston, John Robert, London; Ramsay, Henry Ward, St. Mary's; Renny, Eustace George, St. Thomas's; Reynolds, Frank Ernest, London; Rice, Charles Cracroft, St. George's; Robinson, Arthur, Leeds; Simpson, George Albert, London; Sisson, Herbert, London; Smith, Sidney Calvin, Middlesex; Steward, Edward Simmons, Leeds; Stokes, Thomas Pinfold, Sheffield; Stott, John, Manchester; Sturges, Arthur Blackwell, Leeds; Thomas, Cadogan, Middlesex; Wales, Herbert John Leavis, Guy's; Winter, Lawrence Amos, St. Bartholomew's; Withers, Frederick Ernest, St. Bartholomew's; Wood, Cyril George Russ, Bristol; Wylie, James Thomson Roy, St. George's.

PART II.—MATERIA MEDICA AND PHARMACY.

WEDNESDAY, OCTOBER 19, 1887.

QUESTIONS.

1. Give the composition, strength, and dose of the several official preparations containing Arsenium.
2. What is Ergot? Describe its action, and give its official preparations, with the dose of each.
3. Enumerate the alkaloids derived from Cinchona Bark, and name their official salts. What are the preparations of Quinine, and what is its medicinal action?
4. What are the physical properties of Sulphate of Zinc and Sulphate of Magnesium? Give the action and dose of each, and the official preparations of the latter.
5. What are the differences between an Infusion, a Decoction, a Mixture, and a Liquid Extract?
6. What is the most important active principle of each of the following drugs: Rhubarb, Jalap, Nux Vomica, Ipecacuanha, Jaborandi, Conium, Hyoscyamus, and Catechu?

The following are the names of those who passed: Adams, William Francis, King's College; Allott, John Ernest Cecil, Sheffield; Baker, Nugent Brander, St. Bartholomew's; Bennett, Frank, St. Bartholomew's; Bensley, Edwin Edward, Guy's; Berry, Robert Sewers, St. George's; Bingley, Ernest Horsford, St. Mary's; Bishop, Thomas, Birmingham; Boake, Basil, Dublin; Breen, Adrian Louis, St. Bartholomew's; Bridges, Ernest Chittenden, St. Bartholomew's; Burton, Thomas, Private; Cardinall, Charles Daking, St. Bartholomew's; Carter, Hugh Ronald, St. Mary's; Clarke, Ethelbert Henry, Bristol; Collings, Edward Beresford, Leeds; Colyer, Arthur Reginald, Charing Cross; Cooke, William Henry, Private; Cooper, Arthur Tanner, Private; Cooper, Charles Dudley, University College; Cowen, Henry Morris, Toronto; Dale, Cuthbert Bracy, St. Bartholomew's; Daniel, Robert Napier, St. Thomas's; Davis, William, St. Bartholomew's; Dawson, James Benjamin Hoghton, St. Mary's; De Groot, Stanley H. R. Van Rych, King's College; Duffy, Patrick Joseph, University College; Eames, Edward Thomas Philip, London; Edwards, John Hammerton, St. Bartholomew's; Fearnley, Jonathan, Leeds; Fielden, Frederick Ernest, St. Bartholomew's; Floyd, Stephen George, Guy's; Forde, Thomas Arthur Munro, St. Thomas's; Gillett, George Edward, London; Grant, Charles William, St. Bartholomew's; Graydon, Archibald Thomas, St. Thomas's; Greaves, Thomas Neville, King's College; Handcock, William, Leeds; Hayes, Clyde Longman, Sheffield; Heap, Francis Robert, Bristol; Heriot-Hill

Herbert, Bristol; Hern, George, Private; Hinks, Alfred Grosvenor, St. Mary's; Hopewell, Samuel Prince, London; Houghton, Murtaugh James, Birmingham; Hughes, Herbert Edward, King's College; Hunter, William Yeates, Middlesex; Huththwaite, William Henry Joseph, University College; Jack, Cuthbert Fleming, Charing Cross; Jago, Arthur Tilsed, Guy's; James, Rupert, University College; Jones, George Reginald, Liverpool; Keats, William John Charles, St. Bartholomew's; Knipe, George Frederick, Liverpool; Lees, Charles Archibald, St. Mary's; Leonard Robert Cecil, Bristol; Levick, Henry Driffield, Glasgow; Lewis, Ernest Wool, St. George's; Lilley, George William, Leeds; Lodwidge, William Charrott, St. Mary's; Loos, William Christopher, University College; Lys, George, London; McFarlane, Alexander Rastrick, Glasgow; MacGrath, Edmund John, St. George's; MacLeod, Ewan Cameron, Westminster; McTaggart, Norman Ellis Ellis, St. Bartholomew's; March, Edward Gerald, Guy's; Martin, Charles Rudinge, St. George's; Maw, Henry Solomon, St. Bartholomew's; May, Ernest Augustus, St. Bartholomew's; May, George Ernest, London; Millar, William Heptinstall, St. Thomas's; Morris, Cecil George, Bristol; Morris, Henry Cecil Low, St. Mary's; Norton, Archibald, London; Nunes, Herbert Fitz-Stephen, St. Mary's; Nuttall, Walter Wingfield, St. Bartholomew's; Ord-Mackenzie, Stuart Allan, University College; Orr, Walter Hood, St. Bartholomew's; Peake, George Arthur, Bristol; Pollitt, William Edgar, Leeds; Posnett, Edward, Leeds; Powys, Henry Lionel, London; Rice, Charles Cracroft, St. George's; Riley, Fredk. Ratcliffe, London; Roberts, Hubert William, St. George's; Robinson, Charles St. Hill, St. Mary's; Rock, Frank Warner, St. Bartholomew's; Rodd, Montague Louis Bouchier, Middlesex; St. Stephen, Trewhella, St. Bartholomew's; Scott, Claude Henry, London; Shears, William, St. Bartholomew's; Sheen, Francis Frederick Sydney, Guy's; Smith, Reginald, Manchester; Snell, Sidney Herbert, University College; Spink, John Topham, Leeds; Spray, James Herbert, Leeds; Squibbs, Robert Edward Portbury, Middlesex; Steward, Edward Simmons, Leeds; Taylor, Ashby, Glasgow; Tomlinson, Henry Edward, Leeds; Toulmin, Ernest William, St. Mary's; Turner, Joseph George, St. Thomas's; Visick, Charles Hedley Clarence, University College; Wagstaff, Frank Alexander, Middlesex; Waters, Harry George, private; Watkins, William, London; West, Lionel Frederick, Leeds; Whitford, Robert Scott, private; Wightman, John Prest, St. Bartholomew's; Williams, David Franklin, Liverpool; Winter, Lawrence Amos, St. Bartholomew's; Wintle, Colston, Bristol; Wood, Thomas Jason, University College; Woods, William Jeaffreson, St. Bartholomew's; Wright, Walter Southey, Bristol; Wroughton, Wm. Charles Haultain, St. Thomas's; Wylie, James Thompson Roy, St. George's.

### PART III.—ELEMENTARY PHYSIOLOGY.

Tuesday, October 18, 1887.

QUESTIONS.

1. Name the tissues shown under the microscopes A, B, C. By what characteristics do you recognise them?
2. What are the muscles concerned in Coughing and Sighing?
3. By what conditions is the Coagulation of the Blood accelerated or retarded?
4. What makes the Blood flow onwards in the Veins?
5. What are the conditions favourable to the action of Saliva and Gastric Juice respectively?
6. Trace the course of the circulation from the Right Ventricle to the Right Auricle?

The following are the names of those who passed: Allen, Charles William, St. Mary's; Atkinson, Jackson Arthur, St. Mary's; Barnard, Leonard Girtin, Guy's; Boake, Basil, Dublin; Bour, Leopold François, University College; Braithwaite, Charles Bernard, Guy's; Brooke, Thomas George Percy, Leeds; Collett, Arthur Thomas, University College; Collings, Edward Beresford, Leeds; Cooper, James, St. Bartholomew's; Cornish, Edward, Guy's; Dawes, Charles Edward, London; †De Gebert, J. M. L., Middlesex; Dodd, Henry Noel Nelthorpe, St. George's; Eames, Edward Thomas Philip, London; Ellis, Fred, London; Farnum, Charles Mortimer Stone, King's College; Firth, Henry Edward, Leeds; †Flint, Frederick Sayer, King's College; Franly, Arthur Britten, St. Mary's; Frazer, Ernest Edward, Guy's; Ghose, Benoy Bhushan, University College; Gill, Sutton Dudley, Birmingham; Girdlestone, Howard Edward, St. Thomas's; Haines, Arthur, St. Thomas's; Hanwell, Gerald Lucas, Charing Cross; Hardcastle, Alfred Herbert, Leeds; Hayes, Clyde Longman, Sheffield; Hemming, Charles Harold, Edinburgh; Holden, George Bayne, University College; Hubbard, Arthur Oldham, St. Bartholomew's; Jack, Cuthbert Fleming, Charing Cross; *Jackson, Alfred, Cork; *Kynsey, Herbert Arthur, University College; Le Geyt, Edward, London; Lesur, Marie Paul Armé, St. Bartholomew's; Lockyer, Gerard Edward, Guy's; McKay, John Lumsden, St. George's; Mellersh, Archibald Ewing, University College; Morris, Cecil George, Bristol; Nayler, John William, Birmingham; Paine, Charles, St. Mary's; Pauli, Edwin Henry Churton, Bristol; Pitts-Tucker, Francis Augustus, St. Thomas's; Ransford, John Ernest, Bristol; Rundle, Frank Carlyon, St. George's; *Rutherford, George James, Middlesex; Shore, Pharez Algernon, Birmingham; Sims, John Hedley, St. Thomas's; †Wallis, Sidney Seymour, St. Bartholomew's; *Ward, Martindale Imeson, St. George's; Waring, Herbert Jacob, St. Bartholomew's; West, John Thomas, Birmingham; Willis, Harry Alexander Legge, St. Mary's.

* Passed in Elementary Anatomy only.
† Passed in Elementary Physiology only.

### SECOND EXAMINATION, October, 1887.

PASS LIST.

Andrew, Francis William, St. Bartholomew's; Andrew, Henry, St. Thomas's; *Atkey, Percy James, St. Thomas's; Bale, William Barker, Manchester; Barrs, John Henry, St. Thomas's; Beesley, Albert James, Liverpool; Bernau, Henry Ferdinand, St. Thomas's; Beville, Frederick Wells, St. Thomas's; Blomfield, Alfred Bealy, London; Brooks, Charles, St. Thomas's; Brownlow, Harry Lurgan, St. Bartholomew's; +Brunton, Edward Walter, Charing Cross; +Burditt, Ralph Austin, Manchester; Burton, William Edward, Liverpool; Butler, Charles, St. Bartholomew's; Cannan, David, St. Bartholomew's; *Chater, Arthur Reginald, St. Mary's; Cohen, Isaac, Charing Cross; Colmer, Robert Jacob, London; *Colton, Frank Septimus, University College; Cooper, Ludford, University College; Corner, Albert, St. Bartholomew's; Covey, Edward Alan Rustat, St. Bartholomew's; Cribb, Arthur William Gordon, Middlesex; Crosskey, Roger, Cambridge and St. Bartholomew's; †Crowther, Charles Keith, St. Bartholomew's; Dawson, Bertrand Edward, London; Drake, Ernest Charles, St. Bartholomew's; †Emlyn, Charles Willmore, St. Bartholomew's; Fisher, Thomas Escolme Hervarre, St. Thomas's; Ford, Frank Chubb, St. Bartholomew's; Gann, Thomas William Francis, Middlesex; *Gideon, George Vernon, St. Mary's; Graham, John Henry Porteous, St. Bartholomew's; Green, Arthur Robert, Birmingham; Gregar, Charles Granville, Guy's; Griffiths, Gilbert Henderson, Liverpool; Handfield-Jones, Charles Ranald, St. Mary's; †Hayford, Ernest James, St. Thomas's; Head, Arthur Horsford, London; *Heaton, Arthur Frederick, St. George's; Hemingway, John, St. Thomas's; †Henning, Thomas Irwin, Galway; Hicks, John Abernethy, Westminster; *Jackson, Fox Turnor, Liverpool; Jacobsen, George Oscar, St. Bartholomew's; +James, Lewis Leon, King's College; Jones, George, London; +Jones, Henry John Rutherford, London; Jordan, John Furneaux, Birmingham; Keeling, Arthur Gadsby, St. Thomas's; Kershaw, Herbert Warren, St. Mary's; †Knapp, Montague Henry, Cork; †Lake, Reginald Willie, University College; Lawrence, John, St. Bartholomew's; Lonnon, Frederick, Charing Cross; Lucas, Arthur, Manchester; Leeder, Forrest Bertram, University College; Madge, Arthur Ernest, London; †Meyer, William Robert, King's College; Mitchell, Frank William Drew, Dublin; Morris, Henry Gibbins, Middlesex; †Morris, James John Nixon, King's College; †Morton, Thomas William, St. Thomas's; Mulvany, Thomas Edward, London; †Nelson, Ernest Magson, Leeds; Norris, Benjamin Stuart, Birmingham; Orford, Robert James, Westminster; *O'Sullivan, Carroll, London; Parbury, Walter Key, St. Bartholomew's; †Paterson, George William, St. Mary's; Pooler, Harry William, Birmingham; Powell, Charles Martin, St. Bartholomew's; Prince, John Woolnough George, Charing Cross; Pritchard, William Briggett, Manchester; Ralph, Charles Hughes Danson, Guy's; Reynolds, Austin Edward, University College; Rix, Francis William, Westminster; *Roberts, John Henry, Guy's; Rogers, Arthur Boolds, Birmingham; †Rooke, Ernest Morley, Guy's; Rygate, Henry Bertram, Guy's; *Sanders, Horace, Charing Cross and Mr. Cook's; Segundo, Charles Sempill de, St. Bartholomew's; Sellers, Sanders, Manchester; Slater, Howard, University College; Smith, Arthur Hopewell, Charing Cross; †Smith, John Wilde, Manchester; †Shackleton, Frederick Percy, Leeds; †Shepherd, Henry Brooks, Middlesex; *Spurrell, William Dewing, Guy's; Stephens, Henry Woolcott, St. Bartholomew's; Taylor, John, Manchester; †Taylor, William, Bristol; *Turner, Felix, University College; *Vallance, Herbert, London and Mr. Cook's; Van Leent, Henry James, Guy's; Vernon, Henry Alfred, London; †Walter, Richard Arthur, St. Bartholomew's; Whistler, James Arthur, St. George's and Mr. Cook's; Wiggins, Henry, Charing Cross; †Williams, Charles, King's College; Wright, Walter Southey, Bristol.

* Passed in Anatomy only. † Passed in Physiology only.

### AMUSEMENTS WITH PROFIT FOR ALL AGES.

**Rules.**—All answers must be addressed to the PRIZE EDITOR, THE HOSPITAL, Norfolk House, Norfolk Street, Strand, W.C., not later than the *first* post on Friday next. The full name and address must in all cases be given, but a *nom de plume* may be added for publication. Only one side of the paper must be written on.

No one will be permitted to win the Monthly or Quart erly Prizes twice in any one year, the annual prizes being offered especially to prevent this.

### FOR MEN AND WOMEN.

### SPECIAL PRIZES.

Not more than fifteen competitors have entered, so the conditions for the two special prizes have not been fulfilled. We have the promise that fifty competitors at least will compete in future. We have, however, decided to award the prize of one guinea to the correspondent who sent in the names of the greatest number of subscribers who agree to enter for the prizes. Condorcet, Miss C. Montgomery, Sherwood House, 26, Pembroke Gardens, W., is the winner, she having sent in the names of fourteen new subscribers.

### WHAT IS DESIRED.

After much consideration we have accepted the view of a majority of our readers who state that the acrostics, though admirably constructed, are beyond the compass of the average reader, owing to the fact that many of them have to move about, and that they have not within reach the books of reference to enable them to hunt up all the lights each week with facility. Many ladies object, with reason, to the algebraical puzzles, as being Greek to them, and beyond their powers altogether. It is further urged that several members of the same family would compete if the coupon were abandoned, and we have therefore decided, as an experiment, to retain it only for the Children's Corner. We propose to substitute the following three classes of prizes as an experiment during the next six months :—

### I.—The Quilter Prizes. Six Guineas.

Mr. Cuthbert Quilter, M.P., being much interested in the voluntary principle of Hospital support has placed certain funds at our disposal, as already announced, which we shall expend upon a plan which, we hope, will materially aid the scheme proposed for helping the Hospitals, published on page 91 of this issue. Condorcet's experience shows that those who are really interested in the Hospitals can help them materially, if they will, by making their work widely known amongst their friends and neighbours.

### For best Specimens of Work.

Three prizes of three guineas, two guineas, and one guinea, will be given to the competitor who sends in the first, second, and third best specimens of work of any kind, suitable for presentation to the inmates of the Hospitals, on or before the 31st of December next. This work may take the form of a toy, a doll, or a scrap book for the children ; an article of clothing, a painting or watercolour, an illuminated card or any other gift, for men or women. It is proposed to arrange for the distribution of the articles sent in for this competition amongst the patients in the hospitals.

### An Order of Associated Hospital Workers.

We propose then to institute an Order of Associated Hospital Workers, whose names and addresses will be published in THE HOSPITAL as they enrol themselves from week to week, who will be supplied with a badge, and be eligible to join The Hospitals Association as associates or members, according to the zeal and success which they bring to bear upon their work for the hospitals. At the present time the Hospitals depend for support upon the contributions of the relatively few, and it is essential to the success of the voluntary principle that in the future the majority of the people shall be interested in them. The Hospitals Association (see page 91) proposes to secure the co-operation and association of all persons interested in dealing with the sick poor, and especially of the clergy and ministers of religion, district visitors, teachers, and boards of guardians. We propose, through the Order of Associated Hospital Workers, with the Aid of the clergy of all denominations, to interest individuals and to secure their co-operation. How this can be done by our readers, and especially by the Clergy, ministers of religion, district visitors, and the friends of Hospitals, we propose to show in this column next week.

### II.—Word and Literary Competition. Three Prizes.

Three prizes of one guinea, 15*s.* and 10*s.* 6*d.* will be given each quarter to the three competitors who extract the largest number of words, derived from the word or phrase set for anatomical dissection. We shall give the newspapers for disection during the first quarter, and commence this week with "THE TIMES."

OBSERVE.—Proper names, abbreviations, foreign words, words of less than four letters and repetitions are barred. Nuttall's dictionary only to be used.

### The Alternative Competition.

Some people hate puzzles of all kinds, including word competitions, but they are fond of correspondence, and many sisters and mothers are continually receiving and sending letters to and from members of the family who are abroad in India or the Colonies. The constant coming and going between England and other portions of the British Empire make it a matter of interest and importance to know something about the life, the climate, the amusements, the adventures, the clothing, and the surroundings of those who seek a livelihood beyond the seas. We shall therefore try to find space for the publication of selected letters from abroad, which may be sent by any of our readers who have friends and relations in foreign lands. We shall give marks for these letters as well as for the word competitions which will count equally for these prizes, so that anyone may enter for both or either, the prizes to be given to those who get the highest number of marks. The first letter from across the seas will be found on page 93 of this issue. It is a record of adventure in Australia, and gives a good idea of one class of letter suitable for this column.

### Entertainments at Hospitals and Asylums.

We propose to give prizes of one guinea and of half-a-guinea respectively to the correspondents who send from time to time between now and the 31st of March next the best, brightest, and briefest descriptions of concerts, theatrical and other entertainments given during the winter to the inmates of any public institution.

NOTE.—No coupons need be sent in connection with any of these competitions, and we hope they are sufficiently varied to induce a large number of persons to enter for them. The Quilter Prizes No. I. will be open until 31st December, 1887. Competition No. II. will end on January 28th, 1888.

### THE CHILDREN'S CORNER.

PRIZES.

FOR MOST CORRECT ANSWERS TO PUZZLES.

This Commenced October 1st, 1887.

One Pound a Month.

FOR BEST LITTLE LETTERS.

Ten Shillings per Quarter.

A PRIZE OF THREE GUINEAS

to the Competitor who shall have sent in the largest number of correct or best answers during the twelve months.

### Second Monthly Competition. Puzzles—First Week.

No. 1. BURIED AUTHORS. I saw Brown in Gateshead yesterday. 2. Near this barn old men sit and tell their tales. 3. My bullfinch Nick eats more than all the canaries.

No. 2. CONUNDRUM. How do we know that the medical profession is the most tedious ?

No. 3. GEOGRAPHICAL ACROSTIC. The initials will give the name of a county in Ireland, and the finals its capital. 1. A town in Cumberland. 2. A city in Portugal. 3. A river in Scotland. 4. A mountain in Switzerland. 5. A very ancient city of Asia Minor.

No. 4. ENIGMA. Perfect with a head, perfect without a head ; perfect with a tail, perfect without a tail ; perfect with either, neither, or both.

### Letters.

Next week, instead of the usual letter subject write an account of the Gunpowder Plot.

### Results of Fourth Week—Puzzles:

No. 12. ARITHMETICAL PUZZLE.

Let 1 = whole number of boys ∴ $\frac{1}{3} + \frac{1}{4} + \frac{1}{5} + \frac{1}{6} = \frac{20 + 15 + 12 + 10}{60} = \frac{57}{60} + 1\text{-}\frac{57}{60} = \frac{3}{60} = \frac{1}{20}$ ∴ $\frac{1}{20} = 6$ other boys ∴ $20 \times 6 = 120$. The total number of boys in the school

No. 13. HISTORICAL PUZZLE. In 1789 France was divided, monarchy obliterated, laws turned upside down, religion set aside, and rebellion reigned in all quarters.

No. 14. CONUNDRUM. The answer to this is veay simple, though it has given rise to a good many clever guesses. The answer is, a man crossing a bridge with a bucket of water on his head.

No. 15. SQUARE WORD.

M E A T
E L L A
A L U M
T A M E

Pepita and Alsie have given Emma as the second light; this cannot be accepted, as a square must read downwards and across the same.

*All right*—Jerusalem, Judy, Joe, Mount Edgecumbe. *Three right*—Paddy, G. B. P., Gem, A. C. K. *Two right*—Skye, Ossian, The Eda-Fern, Scrooge, A. G. S. McCulloch, Tim, Toby, Pepita. *One right*—Poodle, Spider, Alsie, Princess Ida.

### Windsor Castle.

The letters this week on *Windsor Castle* are, with one or two exceptions, unusually good, but I have only space for one to-day.

Three marks each :—Gem, Princess Ida, The Eda. Two marks each—Ossian, Twaddle, Mount Edgecumbe. One mark each—Skye, Poodle.

### Notices to Correspondents.

SPIDER.—All letters must be addressed PRIZE EDITOR, etc.

